# Supplementary material for: Interactions Between Inbreeding, Fitness and the Bacterial Microbiome in Aedes aegypti Mosquitoes
Source: Evol Appl. 2026 Jul 31;19(8):e70308. doi: 10.1111/eva.70308 (PMC13426023; doi:10.1111/eva.70308)
Supplement: Supplementary file 1 — Figure S1: Life history traits of select inbred Aedes aegypti populations at G8. (A) Fecundity, (B) egg hatch, (C) survival to pupa, (D) sex ratio, (E) female development time and (F) male development time. Horizontal lines and error bars show medians and 95% confidence intervals with dots showing data from individual females (A, B) or replicate containers (C–F). Figure S2: Regression between composite fitness and estimates of genetic diversity of inbred Ae. aegypti lines using the (a) Ne(P) and (b) Ne(JR) methods for Ne and (c) the proportion of variable sites from the founding populations that became fixed. Shaded areas represent 95% SE. Figure S3: Relative read abundance of specific taxa for each Ae. aegypti line. Figure S4: Robust Aitchison ordination of bacterial community composition across Aedes aegypti lines. Ordination was performed on robust Aitchison distances calculated based on ASV relative abundance data visualized by PCoA. Each point represents an individual line and is labelled by line ID. Figure S5: Spearman correlation matrix of life history traits, genetic diversity and microbiome composition across Aedes aegypti populations when not corrected for multiple comparisons. Panel (a) shows correlations between the different life history traits as well as between measures of genetic diversity (Ne(P) and proportion fixed sites) and life history traits. Panel (b) shows correlations between life history traits (including composite fitness and genetic diversity) and microbiome traits (number of unique ASVs, Shannon index and the relative abundance of the 25 most common microbial taxa). Panel (c) shows correlations between the relative abundance of different microbes. Purple indicates a significant negative correlation while green indicates a positive correlation, and the circles are sized relative to the associated R 2 value. Only significant correlations (p < 0.05) are shown. These results are presented for exploratory purposes only and should be interpret [file EVA-19-e70308-s001.docx]

**Supporting information**


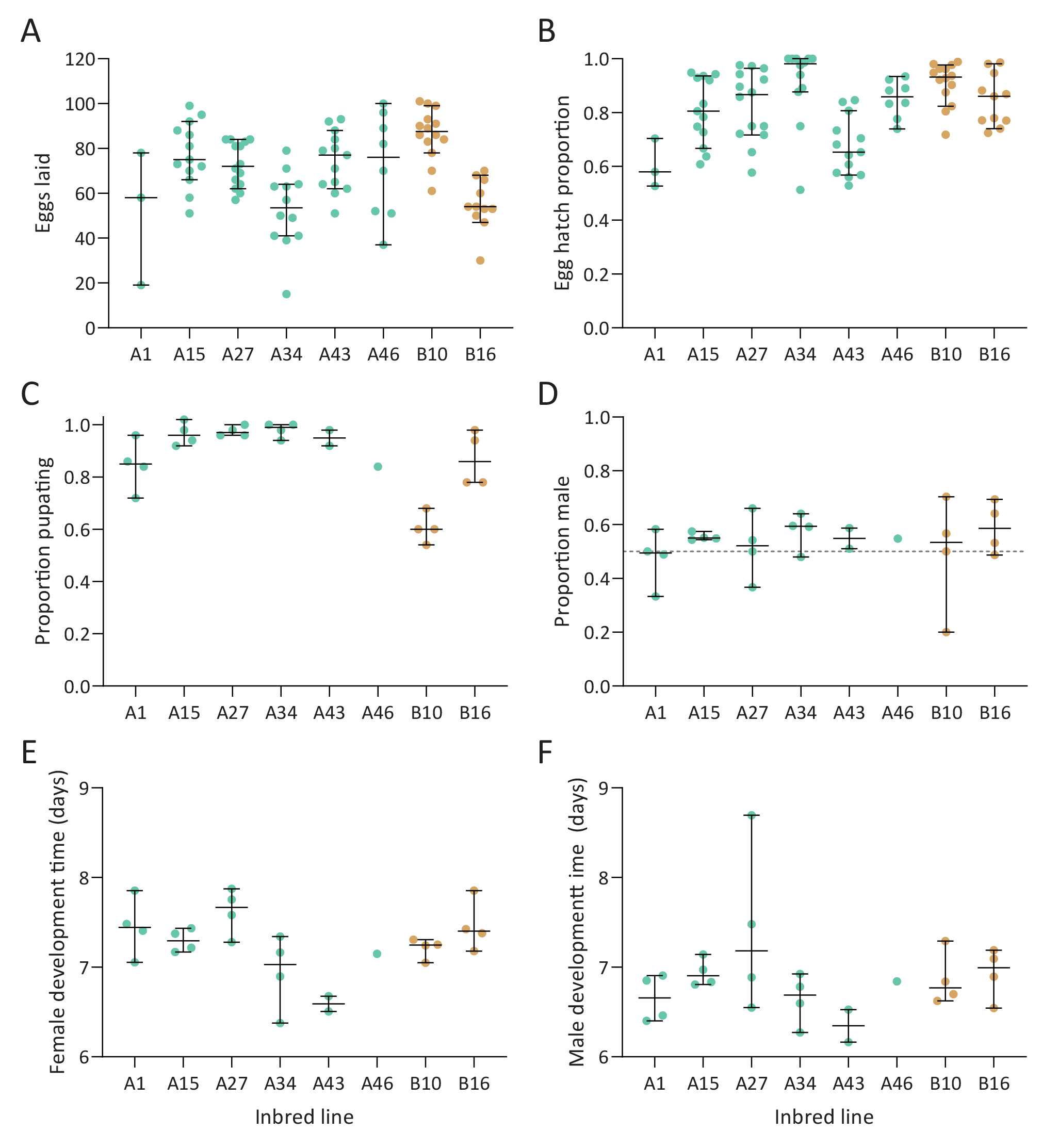


**Figure S1. Life history traits of select inbred *Aedes aegypti* populations at G8.** (A) Fecundity, (B) egg hatch, (C) survival to pupa, (D) sex ratio, (E) female development time and (F) male development time. Horizontal lines and error bars show medians and 95% confidence intervals with dots showing data from individual females (A-B) or replicate containers (C-F).

**
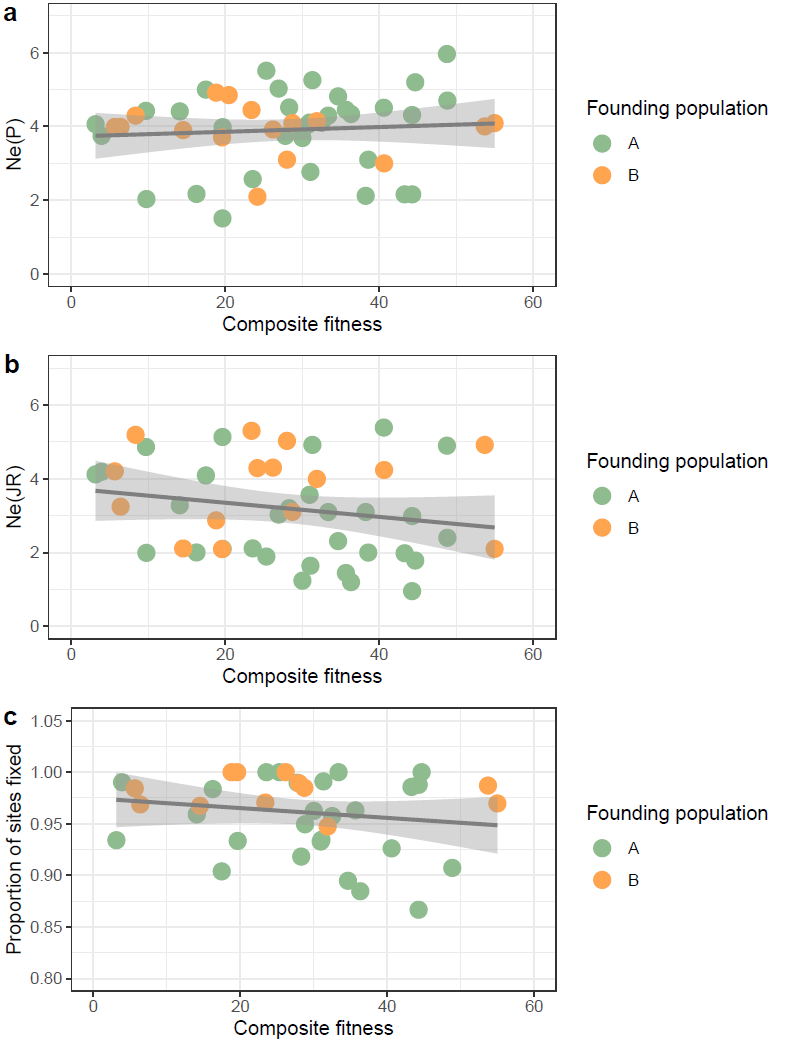
**

**Figure S2. Regression between composite fitness and estimates of genetic diversity of inbred *Ae. aegypti* lines using the (a) N_e_(P) and N_e_(JR) methods for N_e_ and (c) the proportion of variable sites from the founding populations that became fixed.** Shaded areas represent 95% SE.


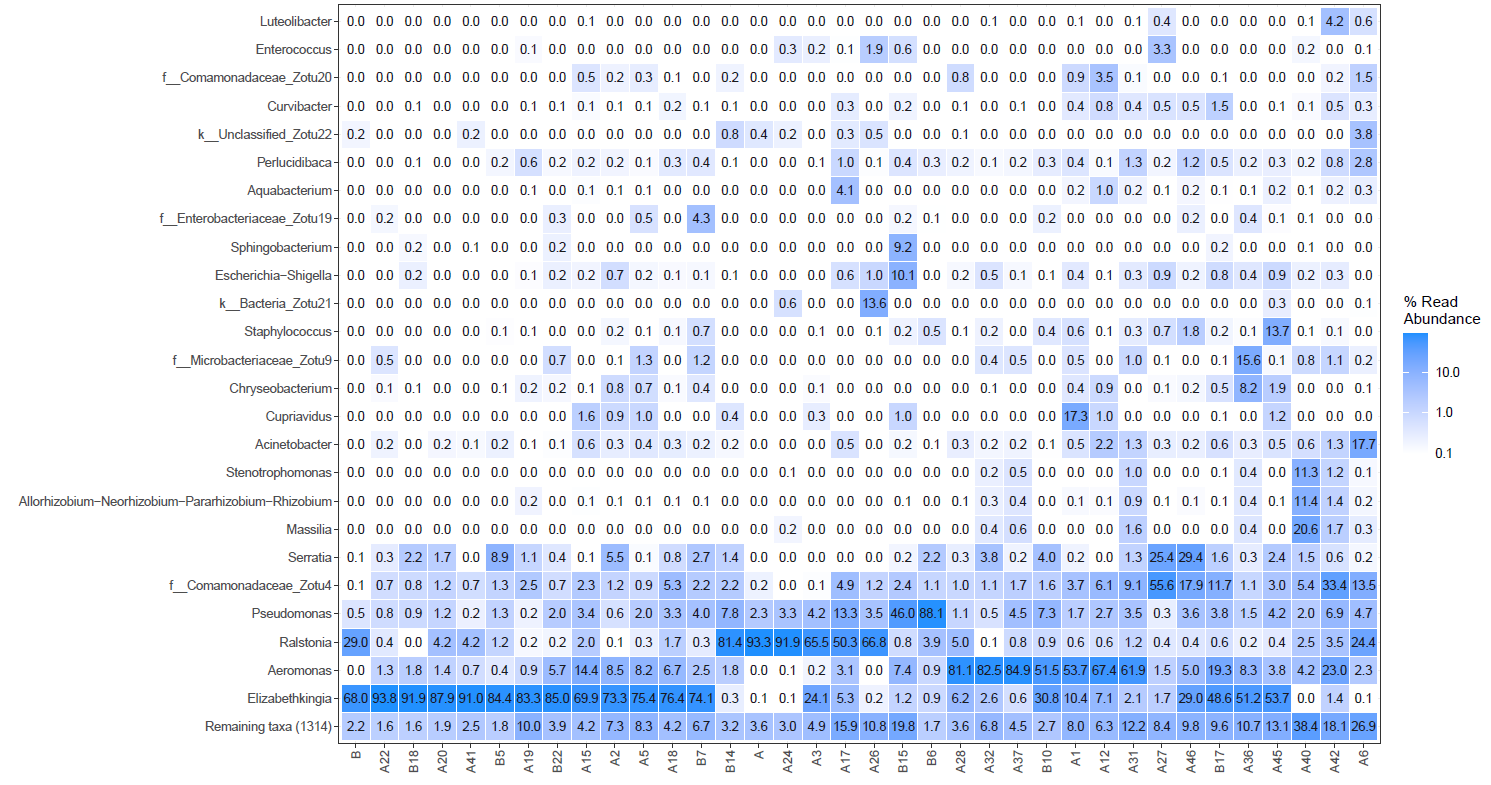


**Figure S3. Relative read abundance of specific taxa for each *Ae. aegypti* line.**


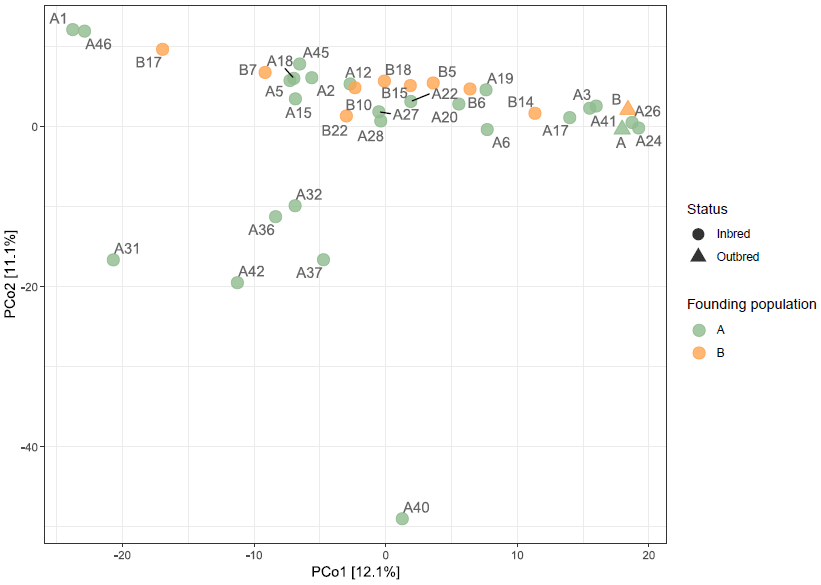


**Figure S4. Robust Aitchison ordination of bacterial community composition across *Aedes aegypti* lines.** Ordination was performed on robust Aitchison distances calculated based on ASV relative abundance data visualized by PCoA. Each point represents an individual line and is labelled by line ID.

**
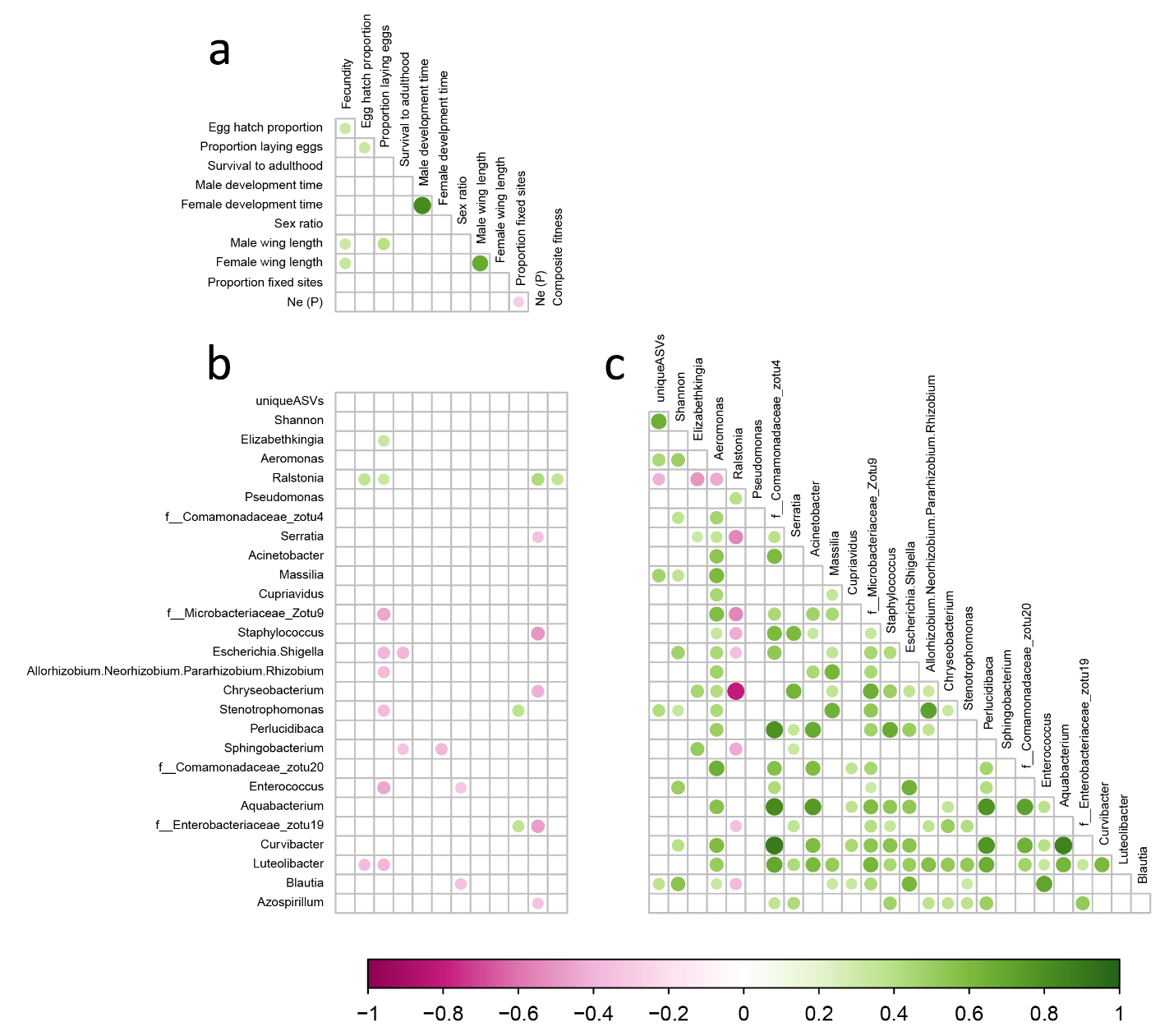
**

**Figure S5. Spearman correlation matrix of life history traits, genetic diversity and microbiome composition across *Aedes aegypti* populations when not corrected for multiple comparisons.** Panel (A) shows correlations between the different life history traits as well as between measures of genetic diversity (Ne(P) and proportion fixed sites) and life history traits. Panel (B) shows correlations between life history traits (including composite fitness and genetic diversity) and microbiome traits (number of unique ASVs, Shannon index and the relative abundance of the 25 most common microbial taxa). Panel (C) shows correlations between the relative abundance of different microbes. Purple indicates a significant negative correlation while green indicates a positive correlation, and the circles are sized relative to the associated R^2^ value. Only significant correlations (P < 0.05) are shown. These results are presented for exploratory purposes only and should be interpreted with caution given the large number of pairwise comparisons performed. Formal inference is based on the multiple-testing-corrected results shown in Figure 2.

**Table S1. ANOVAs for life history traits of select inbred *Aedes aegypti* populations at G8.**

| **Source** | **Degrees of freedom** | **F** | **P value** |
| --- | --- | --- | --- |
| **Fecundity** |  |  |  |
| Intercept | 1 | 1430.132 | < 0.001 |
| Inbred line | 7 | 7.739 | < 0.001 |
| Error | 80 |  |  |
| Total | 88 |  |  |
|  |  |  |  |
| **Egg hatch proportion** |  |  |  |
| Intercept | 1 | 3554.463 | < 0.001 |
| Inbred line | 7 | 7.433 | < 0.001 |
| Error | 80 |  |  |
| Total | 88 |  |  |
|  |  |  |  |
| **Survival to pupa** |  |  |  |
| Intercept | 1 | 3752.986 | < 0.001 |
| Inbred line | 7 | 13.801 | < 0.001 |
| Error | 19 |  |  |
| Total | 27 |  |  |
|  |  |  |  |
| **Sex ratio** |  |  |  |
| Intercept | 1 | 456.380 | < 0.001 |
| Inbred line | 7 | 0.452 | 0.857 |
| Error | 19 |  |  |
| Total | 27 |  |  |
|  |  |  |  |
| **Female development time** |  |  |  |
| Intercept | 1 | 15083.815 | < 0.001 |
| Inbred line | 7 | 4.159 | 0.006 |
| Error | 19 |  |  |
| Total | 27 |  |  |
|  |  |  |  |
| **Male development time** |  |  |  |
| Intercept | 1 | 5026.571 | < 0.001 |
| Inbred line | 7 | 1.517 | 0.221 |
| Error | 19 |  |  |
| Total | 27 |  |  |

**Table S2. ANOVAs for life history traits of inbred *Ae. aegypti* populations at G10.**

| **Source** | **Sum of squares** | **Degrees of freedom** | **F** | **P value** |
| --- | --- | --- | --- | --- |
| **Fecundity** |  |  |  |  |
| Origin | 14403.15 | 1 | 42.1958 | < 0.001 |
| Line (origin) | 103337.96 | 52 | 5.8220 | < 0.001 |
| Error | 214020.51 | 627 |  |  |
|  |  |  |  |  |
| **Egg hatch proportion** |  |  |  |  |
| Origin | 0.0108 | 1 | 0.1826 | 0.6693 |
| Line (origin) | 20.8108 | 52 | 6.7677 | < 0.001 |
| Error | 37.0776 | 627 |  |  |
|  |  |  |  |  |
| **Survival to pupa** |  |  |  |  |
| Origin | 0.0358 | 1 | 7.0281 | 0.0086 |
| Line (origin) | 1.5624 | 53 | 5.7873 | < 0.001 |
| Error | 1.0544 | 207 |  |  |
|  |  |  |  |  |
| **Sex ratio** |  |  |  |  |
| Origin | 0.0768 | 1 | 10.9829 | 0.0011 |
| Line (origin) | 0.9487 | 53 | 2.5596 | < 0.001 |
| Error | 1.4476 | 207 |  |  |
|  |  |  |  |  |
| **Female development time** |  |  |  |  |
| Origin | 13.5811 | 1 | 104.8124 | < 0.001 |
| Line (origin) | 46.1733 | 53 | 6.7234 | < 0.001 |
| Error | 26.8222 | 207 |  |  |
|  |  |  |  |  |
| **Male development time** |  |  |  |  |
| Origin | 7.0728 | 1 | 90.7918 | < 0.001 |
| Line (origin) | 30.2672 | 53 | 7.3308 | < 0.001 |
| Error | 16.1256 | 207 |  |  |
|  |  |  |  |  |
| **Female wing length** |  |  |  |  |
| Origin | 0.0042 | 1 | 0.5030 | 0.4785 |
| Line (origin) | 4.2505 | 53 | 9.5111 | < 0.001 |
| Error | 4.0474 | 480 |  |  |
|  |  |  |  |  |
| **Male wing length** |  |  |  |  |
| Origin | 0.0477 | 1 | 10.1863 | 0.0015 |
| Line (origin) | 1.8512 | 53 | 7.4582 | < 0.001 |
| Error | 2.3650 | 505 |  |  |

**Table S3. Genetic diversity of outbred and inbred *Ae. aegypti* lines at G10.** Effective population sizes were calculated using both the Ne(JR) (Jorde and Ryman, 2007) and Ne(P) (Jonas et al., 2016) methods relative to founding populations at G0. The proportion of sites that were fixed was calculated by extracting all variable sites from the founding populations and calculating the proportion of these that became fixed in the lines at G10. Values with NA could not be calculated due to a low number of callable sites.

| Mosquito line | Ne(JR) | Ne(P) | Proportion of sites fixed |
| --- | --- | --- | --- |
| A | 87.413 | 77.9873 | 0.764 |
| B | 156.7958 | 169.7325 | 0.789 |
| A1 | 4.1 | 4.061973 | 0.934 |
| A2 | 3.2 | 4.516265 | 0.918 |
| A3 | 1.890466 | 5.513831 | 1.000 |
| A4 | NA | NA | NA |
| A5 | 4.0918 | 5.00092 | 0.904 |
| A6 | 1.782195 | 5.199512 | 1.000 |
| A12 | 5.4 | 4.50935 | 0.926 |
| A13 | NA | NA | NA |
| A14 | 1.0 | 4.311752 | 0.867 |
| A15 | 2.4 | 4.703875 | 0.907 |
| A16 | 2.0009 | 2.169054 | 0.984 |
| A17 | 2.3 | 4.816973 | 0.895 |
| A18 | 3.6 | 4.10627 | 0.933 |
| A19 | 3.28115 | 4.410193 | 0.959 |
| A20 | 1.1961 | 4.337088 | 0.885 |
| A21 | 1.234555 | 3.686361 | 0.963 |
| A22 | 1.64023 | 2.767058 | 0.934 |
| A24 | 5.13467 | 1.50546 | NA |
| A25 | 3.0198 | 4.0971 | NA |
| A26 | 4.8635 | 4.424257 | NA |
| A27 | 4.89977 | 5.96701 | NA |
| A28 | 2.098548 | 3.98102 | 0.933 |
| A29 | 1.980276 | 2.158654 | 0.986 |
| A30 | 3.0987 | 2.118168 | NA |
| A31 | 2.98928 | 2.15765 | 0.988 |
| A32 | 4.1923 | 3.745659 | 0.990 |
| A33 | NA | 3.745659 | 0.990 |
| A34 | 1.99817 | 3.09836 | NA |
| A36 | 2.11109 | 2.572016 | 1.000 |
| A37 | 3.09829 | 4.293494 | 1.000 |
| A38 | 1.99028 | 2.030675 | NA |
| A40 | 4.920283 | 5.25724 | 0.991 |
| A41 | 3.0291 | 5.02918 | NA |
| A42 | 1.444085 | 4.452104 | NA |
| A43 | NA | 4.041361 | 0.963 |
| A45 | NA | 4.100556 | 0.957 |
| A46 | NA | 4.041361 | 0.949 |
| B1 | 2.10928 | 3.9018 | 0.967 |
| B5 | 3.10238 | 4.09183 | 0.985 |
| B6 | 5.02938 | 3.09902 | 0.989 |
| B7 | 4.291 | 2.0947 | NA |
| B8 | 4.23948 | 2.99837 | NA |
| B9 | 4.2029 | 3.9873 | 0.984 |
| B10 | 4.92011 | 4.0009 | 0.987 |
| B11 | 5.3029 | 4.44782 | 0.971 |
| B12 | 4.30293 | 3.91827 | 1.000 |
| B13 | NA | NA | NA |
| B14 | 2.873 | 4.91847 | 1.000 |
| B15 | 3.243 | 3.98709 | 0.969 |
| B16 | 3.9982 | 4.14302 | 0.947 |
| B17 | 2.0983 | 4.091076 | 0.970 |
| B18 | NA | NA | NA |
| B20 | NA | 4.851944 | NA |
| B21 | 2.0938 | 3.70384 | 1.000 |
| B22 | 5.193809 | 4.29384 | NA |
